# A Clinic for Mothers

**Published:** 1921-04-02

**Authors:** 


### A Clinic for Mothers.

A new type of clinic for mothers which has just been founded in London by Mr. Roe and his wife, Miss Marie Stopes, D.Sc., is intended to carry into practice the principles governing motherhood advocated in one of Dr. Stopes's popular books on the subject of maternity. A nurse will be in attendance every day, and once a week a woman doctor will attend to give medical advice. "Our object," said Dr. Stopes to a newspaper representative, "is to give working-class mothers the latest scientific knowledge of motherhood, in order to reduce the death-rate among young children and to increase the survival rate. At present there is an appalling ignorance among mothers as to the best means of contributing to the population the greatest number of healthy, happy children. Owing to that ignorance thousands of mothers are reduced to a state of misery and poverty, and married life to them has become a mockery. In far too many cases weak, sickly children follow each other, frequently year after year, and the result is seen in a heavy death-rate, the shortening of the mother's life, and the wastage of millions of pounds by the State and municipalities on asylums, hospitals, homes, and workhouses for dealing with the unfit." It is true that an appalling amount of ignorance on the part of mothers with regard to the dangers and responsibilities of motherhood still exists, but doctors and others whose work lies among the poorer classes know that there has been a great change in recent years, thanks to the spread of education on such matters, the wider publicity given to the subject in the newspapers and press generally, and more especially owing to the establishment of infants' welfare centres in all parts of the country. There are certain considerations, however, affecting the large and important subject of "birth control" which need fuller discussion than can now be given. These we hope to examine more fully in a forthcoming issue of The Hospital.

